# Observations on the Lumbar-Puncture

**Published:** 1906-03-03

**Authors:** 


### OBSERVATIONS ON THE LUMBAR-PUNCTURE.

Dr. N. A. Pashayan [1] has carried out investigations upon the lymphocyte content of the cerebro-spinal fluid in the case of general paresis and some other psychoses. Lymphocytosis of this fluid has been observed in a number of conditions such as syphilis and chronic alcoholism, but special diagnostic value has been attributed to the phenomenon in the case of general paretics and in tabetics. The author's observations were based upon an examination of the fluid of 97 patients, of whom 27 were general paretics of varying types. Among the latter a marked lymphocytosis was found in 16 instances, in five it was moderate, in three doubtful, and in three absent. In two of the negative cases the disease was active, while the third patient was in a phase of prolonged remission. The type or stage of the disease did not seem to have any special bearing on the degree of lymphocytosis. The highest count was met with in the case of a stuporous dement in good physical health, while a considerable increase occurred both in a patient in a stage of continuous agitation bordering on delirium and in a patient enjoying splendid physical health whose mental symptoms were limited to slight amnesia of recent events with some morbid euphoria and boastfulness.

Of the other diseases represented in the category of observation major epilepsy (24 cases) showed no lymphocytosis except in the case of one patient. This man evidenced a slight increase of lymphocytes, although no evidence was forthcoming of any associated organic disease. Infantile cerebral palsy with epilepsy (two cases), primary lateral sclerosis with imbecility (two cases), hebephrenia (13 cases), katatonia (eight cases), maniacal depressive insanity (seven cases), chronic alcoholic deterioration (seven cases), and paranoiac states (seven cases) all failed to show any lymphocytosis.

The technique of the operation for the withdrawal of the fluid consisted of a preliminary injection of 30 minims of a 4 per cent. solution of cocaine beneath the skin. Ten minutes later the puncture was made between the first and second lumbar vertebræ close to the spinous processes. The position chosen for the patient was recumbency upon the right side, the head and knees being approximated on the chest. It was observed that although the position of all the patients at the moment of puncture was practically identical, the degree of tension of the cerebro-spinal fluid was very variable, although there was no constant relation between the tension of the fluid and the nature of the psychosis. Paretics as a class presented the highest tensions, but there were marked exceptions to the rule. Epileptics and precocious dements showed a high tension, but in the case of the former very low tensions were also met with. A comparative examination of the leucocytes present showed that the polymorphonuclear variety were never present, and that some 80 per cent. of the lymphocytes were large.

As regards the effect of the operation it was noted that certain of the patients became pallid, that the pulse was liable to acceleration and irregularity, and, in particular, that a genuine dyspnœa was a not uncommon event. But no evil effects of any permanent moment marked the proceeding.

[1] Med. Rec., Feb. 10, 1906.

